# Comparison of the Microbiota and Inorganic Anion Content in the Saliva of Patients with Gastroesophageal Reflux Disease and Gastroesophageal Reflux Disease-Free Individuals

**DOI:** 10.1155/2020/2681791

**Published:** 2020-05-13

**Authors:** Elvira E. Ziganshina, Ildar I. Sagitov, Ramilya F. Akhmetova, Gulshat T. Saleeva, Andrey P. Kiassov, Natalya E. Gogoleva, Elena I. Shagimardanova, Ayrat M. Ziganshin

**Affiliations:** ^1^Department of Microbiology, Institute of Fundamental Medicine and Biology, Kazan (Volga Region) Federal University, Kazan 420008, Russia; ^2^Department of Orthopedic Dentistry, Kazan State Medical University, Kazan 420012, Russia; ^3^Department of Morphology and General Pathology, Institute of Fundamental Medicine and Biology, Kazan (Volga Region) Federal University, Kazan 420008, Russia; ^4^Laboratory of Extreme Biology, Institute of Fundamental Medicine and Biology, Kazan (Volga Region) Federal University, Kazan 420021, Russia

## Abstract

The oral cavity is one of the most complex microbial environments; however, the complex nature of the salivary microbiota and the level of inorganic anions in the saliva of subjects with and without gastroesophageal reflux disease (GERD) are poorly understood. The primary goals of this pilot research were to assess differences in salivary bacterial community composition and inorganic anion concentrations between patients with GERD and GERD-free people. Thus, the salivary microbiota within both groups was dominated by these genera: Streptococcus, Prevotella, Porphyromonas, Veillonella, Neisseria, Haemophilus, Fusobacterium, Rothia, and Leptotrichia. However, the relative abundances of the genera Actinomyces, Atopobium, Stomatobaculum, Ruminococcaceae_[G-2], Veillonella, and Leptotrichia were significantly higher in the saliva samples of patients with GERD, while the genera Porphyromonas, Gemella, Peptostreptococcus, and Neisseria were less abundant in this group. The concentrations of chloride, phosphate, and sulphate ions in the human saliva varied among all subjects and sampling time. These results broaden our knowledge of the salivary microbial community composition and chemistry of saliva of patients with GERD and GERD-free individuals.

## 1. Introduction

The oral cavity is one of the most complex microbial environments colonized by a set of microorganisms that play an essential role in maintaining oral homeostasis [1]. However, current evidence demonstrates that oral microbiota is also closely linked to various oral diseases, including periodontitis, peri-implantitis, and dental caries [2, 3], and potentially to systemic diseases, including diabetes, cardiovascular disease, osteoporosis, respiratory diseases, rheumatoid arthritis, and certain cancers [4, 5]. Therefore, a deep understanding of the pathogenic mechanisms that influence the development of various diseases is extremely important.

The development of oral biofilms is affected by different factors, including environmental and behavioral factors and the host's immune response to colonization by microorganisms. Therefore, the immune factors of the host, especially those detected in saliva, can affect susceptibility to oral diseases. Saliva contains a mixture of innate antimicrobial proteins and adaptive immune mediators, which can have a significant impact on the microbial colonization of the human mouth [6]. In addition to the innate antimicrobial proteins and adaptive immune mediators, several organic acids and inorganic ions exist in the oral fluid, which influence on the oral environment [7]. Organic acids are formed during bacterial fermentation, which leads to a decrease in the pH of saliva, establishing a suitable environment for the dissolution of dental minerals and dentine. The solubility of enamel also depends on the concentrations of calcium and phosphate ions in the human saliva or dental plaque [8, 9]. Fluoride in saliva and dental plaque is important in preventing dental caries mostly through remineralization of tooth surfaces [10]. Some other inorganic anions, such as chloride and sulphate, are also critical components of the human saliva [11]. Therefore, the analysis of anions in saliva samples in combination with salivary microbiota is required to foresee the extent of tooth decay caused by bacterial metabolism.

Gastroesophageal reflux disease (GERD) is one of the most common and controlled diseases in primary and secondary care settings and is a condition in which the content of the stomach flow back into the esophagus and oral cavity. This usually occurs as a result of the weakening of the muscle ring at the bottom of the esophagus. GERD has a high prevalence in the world (10–40% of the population of various countries). Nocturnal GERD is characterized by acid reflux into the esophagus during sleep. Within many symptoms of GERD, heartburn, regurgitation, unexplained chest pain, chronic cough, difficult or painful swallowing, and asthma can be distinguished. Dental erosion by stomach acids may be another sign of GERD in people who have not yet experienced typical symptoms [12, 13]. Such manifestations of the disease can also influence on the oral microbial community structure [14, 15]. However, despite the importance of oral microbiota analysis, little attention has been paid to the characterization of the salivary microbiota in people with gastroesophageal reflux disease, which might affect the microbial community structure. To date, additional research should be performed to compare the salivary microbiota of GERD patients with that of GERD-free individuals. In addition, in the context of individualized medicine, it is very important to identify changes in an oral bacterial community that are related to host organism genetics and lifestyle factors.

The main goal of this pilot study was to use a bacterial 16S rRNA gene amplicon sequencing approach to receive a complete picture of the salivary microbiota of people with GERD and GERD-free individuals as well as to explore the variability in salivary microbiota profiles in the Russian population. Other goals of this research were to determine the level of inorganic anions in the human saliva and compare salivary inorganic anion concentrations in individuals with and without GERD.

## 2. Materials and Methods

### 2.1. Patients and Inclusion Criteria

In our study, all participants (57% men and 43% women) were diagnosed according to established criteria. The group of patients consisted of fourteen individuals and included patients with GERD. The control group comprised twelve GERD-free participants. Verification of gastroesophageal reflux disease was based on a comprehensive clinical study using various methods to assess the functional state of the main analyzers responsible for the formation of the symptom complex of reflux esophagitis. To verify GERD, we used clinical research methods, including esophagogastroduodenoscopy.

The persons participating in this study were selected from a pool of patients referred to Kazan State Medical University (Kazan, Russia) in summer, 2017. All people that participated in this study were not registered at other health facilities. In general, women were not pregnant and all individuals did not have type I or type II diabetes, coronary heart disease, rheumatic diseases, lupus erythematosus, and HIV infection and did not use antibiotics for 3 months prior to the study.  Table 1 shows the demographic and clinical characteristics of patients with GERD and GERD-free controls. Categorical variables were plotted in contingency tables and estimated by using the Chi^2^ test and Yates continuity correction. Continuous data for normal distribution were tested using the Kolmogorov-Smirnov test. Student's *t*-test for normally distributed values was then used.

The saliva of people with GERD (rd) and GERD-free individuals (hc) was collected without prior stimulation in 15 mL sterile Falcon tubes. Symptoms of reflux are sometimes more noticeable at night, because the stomach acids enter the esophagus more easily when patients are in a horizontal position. Therefore, saliva samples (~2 mL) were collected in the early morning before the personal hygiene of the oral cavity on an empty stomach. Participants were asked not to eat and drink for, at least, 8 h and not to brush their teeth with dental paste in the morning before sampling. In total, three samples (s1, s2, and s3) were collected from each subject with a time interval of 4–5 days. Within an hour, the saliva samples were centrifuged at 10,000×g and then separated into liquid and solid phases. The resulting liquid portion was additionally filtrated through disposable syringe filters (pore size, 0.2 mm) and then analyzed. The precipitated saliva samples were used for DNA extraction and oral microbial community structure analysis. To study the pH level of the gastric juice, each patient underwent esophagogastroduodenoscopy, which was used to collect gastric juice in the amount of 2–3 mL in sterile biomaterial containers (in total, one sample was collected).

The studies were conducted in accordance with approved guidelines for experimentation involving human subject. In accordance with the requirements of the Ethical Committee of Kazan Medical State University (Kazan, Russia), written informed consent was obtained from all persons studied here.

### 2.2. Microbial DNA Extraction and PCR Amplification

Total DNA from saliva samples (solid phases) was extracted using a FastDNA spin kit (MP Biomedicals, USA) and a FastPrep-24 homogenizer (MP Biomedicals, USA) according to the protocol provided by the manufacturer. The extracted DNA was quantified with a NanoDrop 2000 spectrophotometer (Thermo Fisher Scientific, USA) and further used as a template for PCR. Bakt_341F (5′-CCT ACG GGN GGC WGC AG-3′) and Bakt_805R (5′-GAC TAC HVG GGT ATC TAA TCC-3′) were used to amplify V3 to V4 variable regions of the bacterial 16S rRNA gene. Each sample was amplified in triplicate (25 cycles) and prepared for sequencing as described previously [16, 17]. A negative control with sterile MQ water obtained during DNA extraction confirmed the absence of contamination. The final libraries containing 16S rRNA genes were sequenced by a HiSeq 2500 Sequencing System (Illumina, USA) at Joint KFU-Riken Laboratory, Kazan Federal University (Kazan, Russia). 16S rRNA gene amplicon sequencing for each sample was conducted in duplicate to ensure reproducibility (two technical replicates were obtained), and these data are presented as means. Sequence reads of the analyzed samples are available under SRA accession: PRJNA598080.

### 2.3. Microbial Data Analysis

Obtained data were further analyzed using the bioinformatics pipeline QIIME, version 1.9.1 [18], as described previously [16, 17]. Taxonomy to the OTUs (97% identity threshold) was assigned based on the best match to the reference sequences from the Human Oral Microbiome Database [19] and according to the Ribosomal Database Project [20]. OTUs representing <0.1% of the total 16S rRNA reads were also eliminated. Alpha diversity was assessed on an OTU level using the QIIME scripts for calculating Chao1, Shannon, and Simpson indices. Continuous data for normal distribution were tested using the Kolmogorov-Smirnov test. Alpha diversity comparisons and differences in the relative abundances of taxa in two group samples were analyzed using the Mann-Whitney *U* test and Kruskal-Wallis test (nonnormally distributed values). *P* values < 0.05 were considered to indicate statistical significance. Correlations between anions levels were analyzed with the RStudio (based on Spearman's rank correlation coefficient).

### 2.4. Analytical Methods

To analyze the content of anions, samples of filtered saliva were diluted 20 times with MQ water. Chloride, phosphate, and sulphate concentrations in samples were determined using a Thermo Scientific Dionex ICS-900 Ion Chromatography System equipped with a conductivity detector, an IonPac AG22 (4 × 50 mm) guard column, and an IonPac AS22 (4 × 250 mm) analytical column. The mobile phase consisted of 4.5 mM Na_2_CO_3_/1.4 mM NaHCO_3_ at a flow rate of 1.2 mL min^–1^. Data acquisition and instrument control were performed with the Dionex Chromeleon software. pH was measured with a Starter 5000 pH meter and STMICRO8 electrode (OHAUS Corporation, USA). For the statistical evaluation of these data, Mann-Whitney *U* Test or Kruskal-Wallis Test were used (nonnormally distributed values). *P* values < 0.05 were considered to be significant.

## 3. Results

### 3.1. Demographics and Clinical Characteristics

We performed a study to evaluate possible differences in the bacterial composition and inorganic anion content in the saliva between patients with gastroesophageal reflux disease and reflux disease-free controls. The demographic and clinical characteristics of the subjects involved in this study are demonstrated in Table 1. No significant differences were found for patients with GERD and GERD-free controls in relation to age, gender, smoking behavior, number of teeth, and toothbrushing frequency.

### 3.2. Microbiota Structure Analysis

A total of 6.3 million high-quality sequences without chimeras were obtained by investigating 76 saliva samples (26 subjects, 3 samples per subject), and the average number of reads per sample was 82,568 (ranging from 52,969 to 109,545). In the case of hc08s3 and rd11s3 samples, the received data were eliminated from the downstream analysis because of the low sequence counts. Alpha diversity indices calculated on the OTU level are demonstrated in Figure S1 (Supplementary Information). The number of OTUs in the saliva of GERD-free controls ranged from 75 to 105, and the number of OTUs in the saliva of patients with GERD varied from 74 to 108 (abundance > 0.1%). We also measured the Chao1, Shannon, and Simpson indices of individual microbiota. However, there were no statistically significant differences in the Chao1, Shannon, and Simpson indices between the two study groups (Figure S1, Supplementary Information).

**Figure 1:**
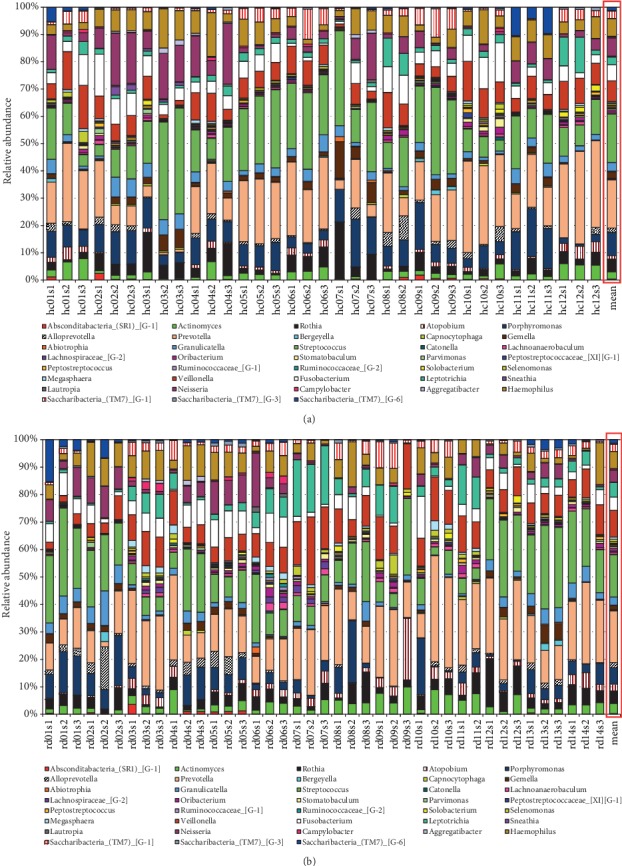
Taxonomic composition of the salivary bacterial communities for (a) GERD-free subjects and (b) patients with GERD. Bacterial community composition according to the sequencing of bacterial 16S rRNA gene is shown on the genus level. In the framed red box, a summary (mean values) of bacterial communities associated with GERD-free controls (hc) and patients with GERD (rd) is demonstrated.

**Figure 2:**
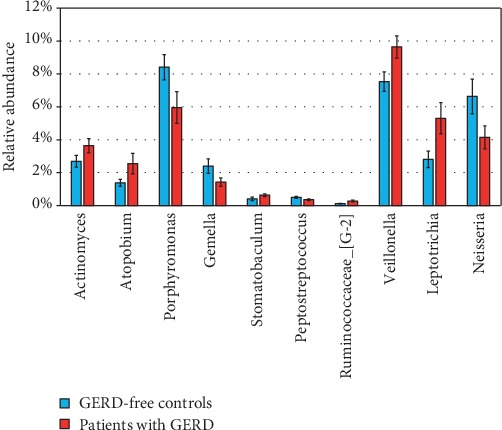
Differentially abundant taxa between GERD-free subjects and patients with GERD. The genera differed in terms of relative abundance (Kruskal-Wallis test; *P* < 0.05). The bars indicate mean ± SEM.

The patient-specific profile of the bacterial community based on the class level is demonstrated in Figure S2 (Supplementary Information). In total, 7 phyla, 14 classes, 17 orders, 25 families, and 38 genera were identified in the saliva samples. The most identified classes in the saliva of subjects within both groups were Bacteroidia, Bacilli, Fusobacteriia, Negativicutes, Actinobacteria, Betaproteobacteria, and Gammaproteobacteria, which accounted for 88.3% of the total bacterial 16S rRNA gene sequences. The salivary microbiota was dominated by these genera: Streptococcus, Prevotella, Porphyromonas, Veillonella, Neisseria, Haemophilus, Fusobacterium, Rothia, and Leptotrichia (Figure 1). These genera accounted for 75.1% of all sequences. No other genera had a relative abundance > 4%. The differences in the overall composition of the salivary microbiota of patients with GERD and GERD-free persons were additionally investigated. The relative abundances of the genera Actinomyces, Atopobium, Stomatobaculum, Ruminococcaceae_[G-2], Veillonella, and Leptotrichia were significantly higher in the saliva samples of patients with GERD, while the genera Porphyromonas, Gemella, Peptostreptococcus, and Neisseria were less abundant in this group (Kruskal-Wallis test, *P* < 0.05) (Figure 2).

### 3.3. Inorganic Anion Level in the Saliva and pH of the Gastric Juice

**Figure 3:**
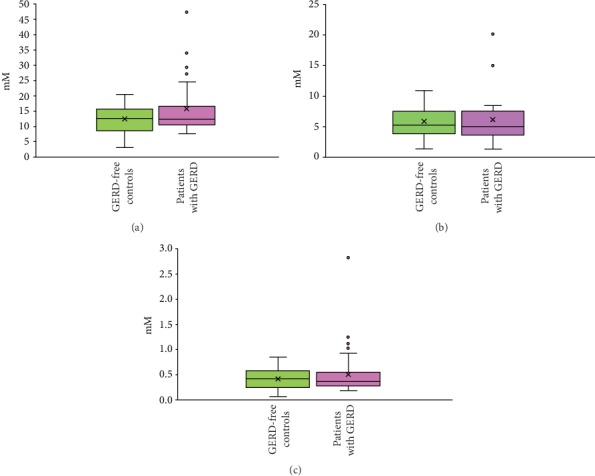
Distribution of chlorides (a), phosphates (b), and sulphates (c) in saliva of GERD-free individuals and patients with GERD.

We further compared the salivary concentrations of chloride, phosphate, and sulphate ions in GERD-free controls to those detected in the saliva of patients with GERD at three different sampling times.  Figure 3 shows the box plots of detected inorganic anion concentrations in the saliva for all study participants, according to established two groups. The mean chloride concentrations in the saliva of controls were lower compared to the mean chloride concentrations in the saliva of GERD patients (12.31 ± 4.59 mM vs. 15.63 ± 8.07 mM, respectively); however, there were no statistically significant differences in these anion levels between the two created groups (*P* = 0.11). In case of other two anions, the values were also not significantly differed between GERD-free controls and patients with GERD, and comparable mean phosphate and sulphate levels were detected in the saliva of people distributed across both groups (5.82 ± 2.66 mM vs. 6.13 ± 4.26 mM and 0.42 ± 0.21 mM vs. 0.51 ± 0.45 mM, accordingly). In addition, independent of sampling time, comparable results were obtained for every person. Nevertheless, notable differences were observed in the salivary inorganic anion concentrations of some patients. For example, several patients contained higher levels of chloride, phosphate, and sulphate ions in their saliva (Figure 3). We additionally analyzed the fluoride concentration in the saliva samples; however, its level was less than the limit of detection in many subjects and were not analyzed further. The pH level was additionally analyzed in gastric juice samples, and comparable mean pH values (~1.9–2.0) were detected in the gastric juice of patients with GERD and GERD-free controls (data not shown).

### 3.4. Correlation Analysis

**Figure 4:**
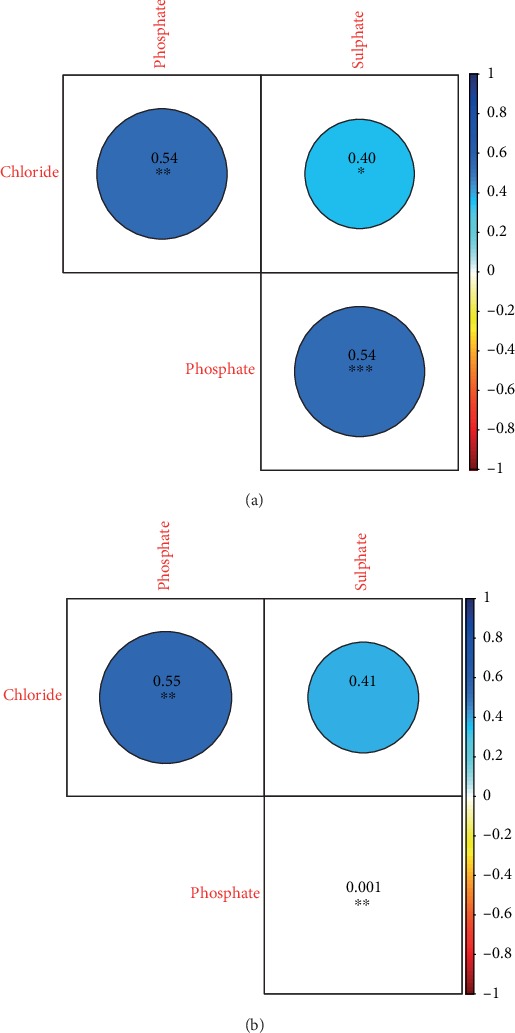
Correlations between inorganic anion levels in the saliva samples of (a) GERD-free subjects and (b) patients with GERD. Spearman's correlation coefficients are shown by color ranging. Negative correlations are displayed in red color, whereas positive correlations are displayed in blue color. Significant correlations are indicated by ^∗^*P* < 0.05, ^∗∗^*P* < 0.01, and ^∗∗∗^*P* < 0.001.

The relationships between salivary inorganic anion concentrations of GERD-free individuals and patients with GERD are demonstrated in Figures 4(a) and 4(b), correspondingly. Thus, the chloride levels were significantly and positively correlated with the phosphate and sulphate concentrations in the saliva of GERD-free individuals (*ρ* = 0.54 and 0.40, respectively). Finally, the correlation coefficient between phosphate and sulphate levels in the control group was 0.54. In the case of the patient group, the salivary chloride concentrations were significantly and positively correlated only with the phosphate concentration (*ρ* = 0.55).

## 4. Discussion

The influence of complex oral microbiota on the health of the host organism has become a subject of interest in recent studies. Many studies assessed differences in the oral microbial composition in patients with various diseases using the next generation sequencing techniques [21–25]. In the research described herein, we used a 16S rRNA gene Illumina sequencing approach to characterize the salivary microbiota of people with gastroesophageal reflux disease, which has not been previously studied well. In addition, we used the ion chromatography system to determine the content of inorganic anions in the saliva of the Russian people with and without gastroesophageal reflux disease.

Gastroesophageal reflux disease happens when the upper part of the digestive tract does not function properly, causing the stomach contents to flow back frequently to the esophagus and oral cavity. Nocturnal GERD is characterized by acid reflux into the esophagus during sleep. Since the gastric acids enter the esophagus more easily when patients are in a horizontal position, symptoms of GERD during night-time sleep are sometimes more noticeable. Heartburn, regurgitation, unexplained chest pain, chronic cough, difficulty swallowing, and asthma are the several symptoms of GERD. Dental erosion by gastric acids can be another sign of GERD in people who have not yet experienced the common symptoms [12, 13]. In individuals with GERD, the gastric content may return to the esophagus and reach the oral cavity leading to dental erosions and carious lesions. Such manifestations of GERD can also impact on the oral bacterial community structure [15]. Therefore, it is imperative to investigate differences in the composition of the microbiota and inorganic anions in the saliva of patients with GERD.

Regardless of the study group, the following bacterial genera prevailed in the human salivary microbiota: Streptococcus, Prevotella, Porphyromonas, Veillonella, Neisseria, Haemophilus, Fusobacterium, Rothia, and Leptotrichia. Genus-level analysis showed that the relative abundances of Actinomyces, Atopobium, Stomatobaculum, Ruminococcaceae_[G-2], Veillonella, and Leptotrichia were higher in the saliva of patients with GERD, whereas Porphyromonas, Gemella, Peptostreptococcus, and Neisseria demonstrated lower proportions. 16S rRNA gene sequences closely related to facultative anaerobic Streptococcus salivarius and Streptococcus oralis, which are pioneer oral bacterial species [26], were detected in the human saliva at high levels. Streptococcus species produce lactic acid as the major terminal product of fermentation of glucose [27], and, therefore, cause a decrease in the pH level of the oral fluid. Several members of the genus Streptococcus are part of the normal human oral microbiota but capable of opportunistic pathogenicity, while others are pathogens [28, 29]. S. salivarius may contribute to the creation of immune homeostasis and the regulation of inflammatory reactions of the body [30]. These early oral cavity colonizers are followed by anaerobes, such as representatives of the genera Prevotella, Porphyromonas, Fusobacterium, and Veillonella [26, 31].

Prevotella species have been connected with several oral infections and are part of the healthy oral microbiota [31, 32]. They can produce various organic acids from some sugars, including acetic acid and succinic acid as the major end acid products [31]. Species of the mostly pathogenic genus Porphyromonas (were less abundant in the saliva of people with GERD), in general, are asaccharolytic bacteria; however, P. pasteri is weakly saccharolytic and can produce moderate amounts of propionic acid and small amounts of acetic, isovaleric, and succinic acids from sugars [33]. 16S rRNA gene sequences closely related to the species P. pasteri were found at high levels in our samples in both groups. Capnocytophaga species are facultative anaerobic bacteria of the normal oral microbiota of humans and considered as opportunistic pathogens [34] that can produce acetic and succinic acids [35]. Bacteria of such genera as Veillonella, Neisseria, Haemophilus, Fusobacterium, Rothia, and Leptotrichia were detected at notable levels in the human saliva of both groups. The relative abundances of the genera Veillonella, Leptotrichia, and Actinomyces were significantly higher in the saliva samples of patients with GERD, while the genus Neisseria was less abundant in this group (*P* < 0.05). They are also part of the normal oral microbiota and can be connected with several oral infections, and most of their representatives can generate various organic acids [36]. This leads to a saliva pH decrease and finally to the degradation of enamel on the teeth and the development of caries [37, 38].

In addition, the relative abundances of Stomatobaculum and Atopobium were significantly higher in the saliva samples of the patient group (*P* < 0.05). Stomatobaculum longum isolated previously from the subgingival plaque of dentally healthy human subject has been reported to ferment various carbohydrates to butyric, lactic, isovaleric, and acetic acids as the major fermentation products [39]. Atopobium parvulum as representative of the genus Atopobium can be isolated from the human oral cavity and is associated with halitosis (as a sign of certain diseases of the human digestive system, accompanied by a pathological increase in the number of anaerobic microbes in the oral cavity and an unpleasant smell from the mouth) [40, 41]. Nevertheless, A. parvulum has also been detected in the saliva of healthy subjects [42] and on the tongue dorsum of individuals without halitosis [43]. It is worth noting that, in addition to A. parvulum, various Prevotella species and S. salivarius have also been detected on the tongue dorsum of subjects with bad breath [43]. Since we sampled the saliva in the early morning before the personal hygiene of the oral cavity, these bacterial species, as well as many others, were probably involved in producing malodorous compounds such as volatile sulfur compounds.

Chloride, phosphate, and sulphate concentrations were next analyzed in the human saliva samples. Despite the absence of statistically significant differences in the mean levels of these anions between both established groups, notable differences were observed in the salivary inorganic anion concentrations of some patients. Thus, some patients had higher concentrations of chloride, phosphate, and sulphate in their saliva. Interestingly, a significant and positive correlation was found between the concentrations of chloride, sulphate, and phosphate in the saliva of only GERD-free individuals. On the other hand, positive correlations were found between chloride and phosphate levels in the saliva of people with GERD. The concentrations of these anions in the saliva observed in this research are consistent with some previous works that focused on the analysis of different anions in unstimulated and stimulated saliva [7, 44]. These authors also observed a strong positive correlation between the concentrations of chloride, sulphate, and phosphate in the human saliva. Except for the low pH of the human saliva, the solubility of enamel also depends on the levels of calcium and phosphate ions in saliva or dental plaque [8, 9]. For hydroxyapatites, as part of the enamel, it is vital that these ions are abundant enough to maintain a supersaturated state of the saliva. The enamel surfaces can be remineralized by precipitation of calcium phosphate minerals [45]. Fluoride ions in saliva and dental plaque are also important since they inhibit demineralization and enhance the enamel remineralization process [10]. Additionally, chloride, phosphate, and sulphate are important components of saliva as buffer solutions; they stabilize the pH of saliva and decrease the solubility of hydroxyapatites [11]. The pH level of the human saliva depends on several factors, including the presence of specific organic acids in the oral environment. In addition, erosion of teeth by stomach acids can also occur in people with GERD [12, 13]. Lactic, acetic, propionic, formic, butyric, and some other organic acids are the major products generated by various bacteria in the oral cavity [7]. Therefore, the microbial community structure should be carefully investigated to explore the variability in salivary microbiota profiles in GERD-free controls and people with GERD.

## 5. Conclusions

In conclusion, in this research, we comprehensively characterized the microbiota and inorganic anion content in the saliva samples of people with gastroesophageal reflux disease, which has not been well-investigated previously. We showed that the bacterial community structure of the salivary microbiota of people with GERD was slightly different from those of people without GERD. Furthermore, the amount of the inorganic anions varied among all subjects and sampling time, with no significant differences in the mean concentration between both established groups. Nevertheless, notable differences were detected in the salivary inorganic anion concentrations of several patients. These data can serve as a starting point for further study of oral microbial communities of people with gastroesophageal reflux disease.

## Figures and Tables

**Table 1:** Demographic and clinical characteristics of GERD-free controls and patients with GERD.

Variables	GERD-free controls (*n* = 12)	Patients with GERD (*n* = 14)	*P* value
Female (%)	50.0	35.7	0.74^*a*^
Age (mean ± SD)	28.2 ± 5.6	27.5 ± 4.9	0.75^*b*^
Current smoker (%)	16.7	28.6	0.80^*a*^
Past smoker (%)	25.0	14.3	0.85^*a*^
Number of teeth	28.4 ± 2.1	28.1 ± 2.0	0.74^*b*^
Toothbrushing frequency (mean ± SD)	1.50 ± 0.5	1.57 ± 0.5	0.73^*b*^

^a^Chi^2^ test with Yates correction: categorical variables. ^b^Student's *t*-test: normal distribution of metric values (mean ± SD).

## Data Availability

Sequence reads of the analyzed samples are available under SRA accession: PRJNA598080.

## Supplementary Materials

Supplementary Materials Figure S1: calculation of the alpha diversity indices for the salivary bacterial communities associated with gastroesophageal reflux disease-free subjects and patients with gastroesophageal reflux disease (A–observed OTUs; B–Chao1 index; C–Shannon index; D–Simpson index). Figure S2: taxonomic composition of the salivary bacterial communities for (A) gastroesophageal reflux disease-free subjects and (B) patients with gastroesophageal reflux disease. Bacterial community composition according to the sequencing of the 16S rRNA gene is shown on the class level. In the framed red box, a summary of salivary bacterial communities associated with gastroesophageal reflux disease-free controls (hc) and patients with gastroesophageal reflux disease (rd) is demonstrated.

## References

[1] Mandel I. D. (1989). The role of saliva in maintaining oral homeostasis. *The Journal of the American Dental Association*.

[2] Zheng H., Xu L., Wang Z. (2015). Subgingival microbiome in patients with healthy and ailing dental implants. *Scientific Reports*.

[3] Yamashita Y., Takeshita T. (2017). The oral microbiome and human health. *Journal of Oral Science*.

[4] Otomo-Corgel J., Pucher J. J., Rethman M. P., Reynolds M. A. (2012). State of the science: chronic periodontitis and systemic health. *Journal of Evidence-Based Dental Practice*.

[5] He J., Li Y., Cao Y., Xue J., Zhou X. (2015). The oral microbiome diversity and its relation to human diseases. *Folia Microbiologica*.

[6] Simon-Soro A., Sherriff A., Sadique S. (2018). Combined analysis of the salivary microbiome and host defence peptides predicts dental disease. *Scientific Reports*.

[7] Park Y. D., Jang J. H., Oh Y. J., Kwon H. J. (2014). Analyses of organic acids and inorganic anions and their relationship in human saliva before and after glucose intake. *Archives of Oral Biology*.

[8] Dawes C. (2003). What is the critical pH and why does a tooth dissolve in acid?. *Journal of the Canadian Dental Association*.

[9] Meyer F., Amaechi B. T., Fabritius H.-O., Enax J. (2018). Overview of calcium phosphates used in biomimetic oral care. *The Open Dentistry Journal*.

[10] Lussi A., Hellwig E., Klimek J. (2012). Fluorides – mode of action and recommendations for use. *Schweizerische Monatsschrift für Zahnmedizin*.

[11] Leung V. W. H., Darvell B. W. (1997). Artificial salivas for in vitro studies of dental materials. *Journal of Dentistry*.

[12] Badillo R., Francis D. (2014). Diagnosis and treatment of gastroesophageal reflux disease. *World Journal of Gastrointestinal Pharmacology and Therapeutics*.

[13] Wilson J. L., Pruett K. L. (2016). Gastroesophageal reflux disease: treating wisely. *North Carolina Medical Journal*.

[14] Corrêa M. C. C. S. F., Lerco M. M., Cunha M. d. L. R. d. S. d., Henry M. A. C. d. A. (2012). Salivary parameters and teeth erosions in patients with gastroesophageal reflux disease. *Arquivos de Gastroenterologia*.

[15] Lukina G. I., Krikheli N. I., Ivannikova A. V., Gioeva Y. A. (2018). Subjective, functional and microbiological parameters of the oral cavity in gastroesophageal reflux patients with acidic and subacidic refluctant. *Stomatologiia*.

[16] Ziganshina E. E., Mohammed W. S., Shagimardanova E. I., Vankov P. Y., Gogoleva N. E., Ziganshin A. M. (2018). Fungal, bacterial, and archaeal diversity in the digestive tract of several beetle larvae (Coleoptera). *BioMed Research International*.

[17] Mohammed W. S., Ziganshina E. E., Shagimardanova E. I., Gogoleva N. E., Ziganshin A. M. (2018). Comparison of intestinal bacterial and fungal communities across various xylophagous beetle larvae (Coleoptera: Cerambycidae). *Scientific Reports*.

[18] Caporaso J. G., Kuczynski J., Stombaugh J. (2010). QIIME allows analysis of high-throughput community sequencing data. *Nature Methods*.

[19] Dewhirst F. E., Chen T., Izard J. (2010). The human oral microbiome. *Journal of Bacteriology*.

[20] Wang Q., Garrity G. M., Tiedje J. M., Cole J. R. (2007). Naive Bayesian classifier for rapid assignment of rRNA sequences into the new bacterial taxonomy. *Applied and Environmental Microbiology*.

[21] Szafranski S. P., Wos-Oxley M. L., Vilchez-Vargas R. (2015). High-resolution taxonomic profiling of the subgingival microbiome for biomarker discovery and periodontitis diagnosis. *Applied and Environmental Microbiology*.

[22] Vankov P. Y., Ziganshina E. E., Ilinskaya O. N., Khafizova F. A., Khafizov R. G., Ziganshin A. M. (2016). Comparative analysis of bacterial communities associated with healthy and inflamed peri-implant tissues. *BioNanoScience*.

[23] Sun B., Zhou D., Tu J., Lu Z. (2017). Evaluation of the bacterial diversity in the human tongue coating based on genus-specific primers for 16S rRNA sequencing. *BioMed Research International*.

[24] Zawadzki P. J., Perkowski K., Padzik M. (2017). Examination of oral microbiota diversity in adults and older adults as an approach to prevent spread of risk factors for human infections. *BioMed Research International*.

[25] Schulz S., Porsch M., Grosse I., Hoffmann K., Schaller H. G., Reichert S. (2019). Comparison of the oral microbiome of patients with generalized aggressive periodontitis and periodontitis-free subjects. *Archives of Oral Biology*.

[26] Nascimento M. M., Faintuch J., Faintuch S. (2019). The oral microbiome. *Microbiome and metabolome in diagnosis, therapy, and other strategic applications*.

[27] Toit M., Huch M., Cho G. S., Franz C., Holzapfel W. H., Wood B. J. B. (2014). The genus Streptococcus. *Lactic Acid Bacteria: Biodiversity and Taxonomy*.

[28] Do T., Jolley K. A., Maiden M. C. J. (2009). Population structure of Streptococcus oralis. *Microbiology*.

[29] Pita P. P. C., Rodrigues J. A., Ota-Tsuzuki C. (2015). Oral Streptococci Biofilm Formation on Different Implant Surface Topographies. *BioMed Research International*.

[30] Kaci G., Goudercourt D., Dennin V. (2014). Anti-inflammatory properties of Streptococcus salivarius, a commensal bacterium of the oral cavity and digestive tract. *Applied and Environmental Microbiology*.

[31] Garrett W. S., Onderdonk A. B., Bennett J. E., Dolin R., Blaser M. J. (2015). 249 – Bacteroides, Prevotella, Porphyromonas, and Fusobacterium species (and other medically important anaerobic gram-negative bacilli). *Mandell, Douglas, and Bennett's Principles and Practice of Infectious Diseases (8th ed.)*.

[32] Könönen E. (1993). Pigmented Prevotella species in the periodontally healthy oral cavity. *FEMS Immunology and Medical Microbiology*.

[33] Sakamoto M., Yamashita Y., Shibata Y., Ohkuma M., Takeshita T., Li D. (2015). Porphyromonas pasteri sp. nov., isolated from human saliva. *International Journal of Systematic and Evolutionary Microbiology*.

[34] Zaura E., Keijser B. J., Huse S. M., Crielaard W. (2009). Defining the healthy "core microbiome" of oral microbial communities. *BMC Microbiology*.

[35] London J., Celesk R. A., Kagermeier A., Johnson J. L. (1985). Emended description of Capnocytophaga gingivalis. *International Journal of Systematic Bacteriology*.

[36] Zhou X., Li Y. (2015). *Atlas of Oral Microbiology: from Healthy Microflora to Disease*.

[37] Baliga S., Muglikar S., Kale R. (2013). Salivary pH: a diagnostic biomarker. *Journal of Indian Society of Periodontology*.

[38] Hans R., Thomas S., Garla B., Dagli R. J., Hans M. K. (2016). Effect of various sugary beverages on salivary pH, flow rate, and oral clearance rate amongst adults. *Scientifica*.

[39] Sizova M. V., Muller P., Panikov N. (2013). Stomatobaculum longum gen. nov., sp. nov., an obligately anaerobic bacterium from the human oral cavity. *International Journal of Systematic and Evolutionary Microbiology*.

[40] Kazor C. E., Mitchell P. M., Lee A. M. (2003). Diversity of bacterial populations on the tongue dorsa of patients with halitosis and healthy patients. *Journal of Clinical Microbiology*.

[41] Copeland A., Sikorski J., Lapidus A. (2009). Complete genome sequence of Atopobium parvulum type strain (IPP 1246). *Standards in Genomic Sciences*.

[42] Downes J., Munson M. A., Spratt D. A. (2001). Characterisation of Eubacterium-like strains isolated from oral infections. *Journal of Medical Microbiology*.

[43] Riggio M. P., Lennon A., Rolph H. J. (2008). Molecular identification of bacteria on the tongue dorsum of subjects with and without halitosis. *Oral Diseases*.

[44] Chen Z. F., Darvell B. W., Leung V. W. (2004). Human salivary anionic analysis using ion chromatography. *Archives of Oral Biology*.

[45] Edgar W. M., O’Mullane D. M. (1996). *Saliva and oral health*.

