# Supplementary material for: Comparison of the Microbiota and Inorganic Anion Content in the Saliva of Patients with Gastroesophageal Reflux Disease and Gastroesophageal Reflux Disease-Free Individuals
Source: Biomed Res Int. 2020 May 13;2020:2681791. doi: 10.1155/2020/2681791 (PMC7244971; doi:10.1155/2020/2681791)
Supplement: Supplementary Materials — Figure S1: calculation of the alpha diversity indices for the salivary bacterial communities associated with gastroesophageal reflux disease-free subjects and patients with gastroesophageal reflux disease (A–observed OTUs; B–Chao1 index; C–Shannon index; D–Simpson index). Figure S2: taxonomic composition of the salivary bacterial communities for (A) gastroesophageal reflux disease-free subjects and (B) patients with gastroesophageal reflux disease. Bacterial community composition according to the sequencing of the 16S rRNA gene is shown on the class level. In the framed red box, a summary of salivary bacterial communities associated with gastroesophageal reflux disease-free controls (hc) and patients with gastroesophageal reflux disease (rd) is demonstrated. [file 2681791.f1.pdf]

Supplementary Information:

Comparison of the Microbiota and Inorganic Anion Content in the Saliva of Patients with Gastroesophageal Reflux Disease and Gastroesophageal Reflux Disease-Free Individuals

Elvira E. Ziganshina,[1] Ildar I. Sagitov,[2] Ramilya F. Akhmetova,[1] Gulshat T. Saleeva,[2] Andrey P. Kiassov,[3] Natalya E. Gogoleva,[4] Elena I. Shagimardanova,[4] and Ayrat M. Ziganshin[1]

[1]*Department of Microbiology, Institute of Fundamental Medicine and Biology, Kazan (Volga Region) Federal University, Kazan 420008, Russia*

[2]*Department of Orthopedic Dentistry, Kazan State Medical University, Kazan 420012, Russia*

[3]*Department of Morphology and General Pathology, Institute of Fundamental Medicine and Biology, Kazan (Volga Region) Federal University, Kazan 420008, Russia*

[4]*Laboratory of Extreme Biology, Institute of Fundamental Medicine and Biology, Kazan (Volga Region) Federal University, Kazan 420021, Russia*

* E-mail: ayrat.ziganshin@kpfu.ru (A.M. Ziganshin).

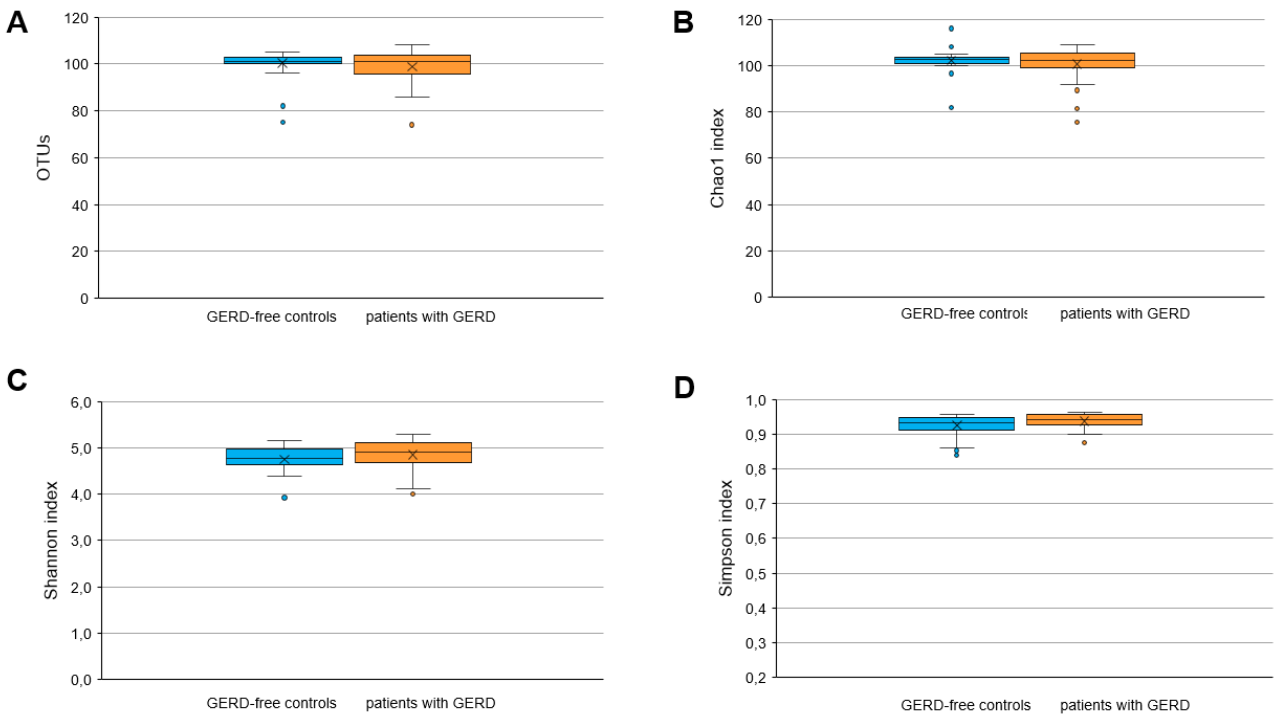

Figure S1. Calculation of the alpha diversity indices for the salivary bacterial communities associated with gastroesophageal reflux disease-free subjects and patients with gastroesophageal reflux disease (A – observed OTUs; B – Chao1 index; C – Shannon index; D – Simpson index).

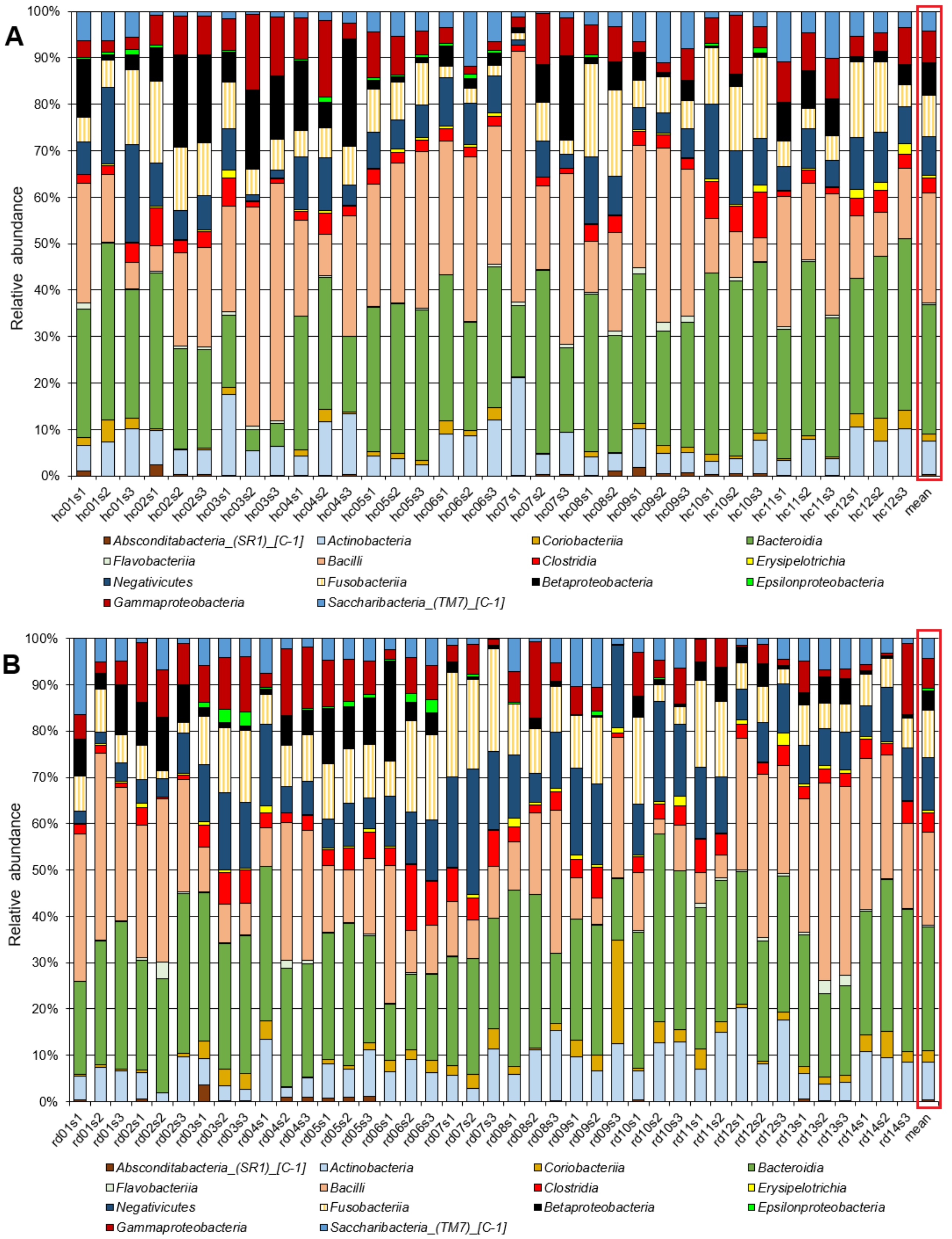

Figure S2. Taxonomic composition of the salivary bacterial communities for (A) gastroesophageal reflux disease-free subjects and (B) patients with gastroesophageal reflux disease. Bacterial community composition according to sequencing of 16S rRNA gene is shown on the class level. In the framed red box, a summary of salivary bacterial communities associated with gastroesophageal reflux disease-free controls (hc) and patients with gastroesophageal reflux disease (rd) is demonstrated.
